# Species Identification and Mycotoxigenic Potential of *Aspergillus* Section *Flavi* Isolated from Maize Marketed in the Metropolitan Region of Asunción, Paraguay

**DOI:** 10.3390/microorganisms11081879

**Published:** 2023-07-25

**Authors:** Juliana Moura-Mendes, Cinthia C. Cazal-Martínez, Cinthia Rojas, Francisco Ferreira, Pastor Pérez-Estigarribia, Nathalia Dias, Patrício Godoy, Jéssica Costa, Cledir Santos, Andrea Arrua

**Affiliations:** 1 Centro Multidisciplinario de Investigaciones Tecnológicas, Universidad Nacional de Asunción, San Lorenzo 111421, Paraguay; jmendes@rec.una.py (J.M.-M.);; 2 Facultad de Ciencias Exactas y Naturales, Universidad Nacional de Asunción, San Lorenzo 111421, Paraguay; 3 Facultad Politécnica, Universidad Nacional de Asunción, San Lorenzo 111421, Paraguay; 4 Facultad de Medicina, Universidad Sudamericana, Pedro Juan Caballero 130112, Paraguay; 5 BIOREN-UFRO Scientific and Technological Bioresource Nucleus, Universidad de La Frontera, Temuco 4811230, Chile; 6 Instituto de Microbiología Clínica, Facultad de Medicina, Universidad Austral de Chile, Valdivia 5090000, Chile; 7 Departamento de Biologia, Instituto de Ciências Biológicas-ICB, Universidade Federal do Amazonas, Av. Rodrigo Otávio Jordão Ramos 3000, Bloco 01, Manaus 69077-000, Brazil; jessicacosta@ufam.edu.br; 8 Department of Chemical Science and Natural Resources, Universidad de La Frontera, Temuco 4811230, Chile

**Keywords:** *Aspergillus flavus*, food safety, MALDI-TOF MS, mycotoxins, mycotoxin co-occurrence, polyphasic approach

## Abstract

*Zea mays* var. amylacea and *Zea mays* var. indurata are maize ecotypes from Paraguay. *Aspergillus* section *Flavi* is the main spoilage fungus of maize under storage conditions. Due to its large intraspecific genetic variability, the accurate identification of this fungal taxonomic group is difficult. In the present study, potential mycotoxigenic strains of *Aspergillus* section *Flavi* isolated from *Z. mays* var. indurata and *Z. mays* var. amylacea that are marketed in the metropolitan region of Asunción were identified by a polyphasic approach. Based on morphological characters, 211 isolates were confirmed to belong to *Aspergillus* section *Flavi*. A subset of 92 strains was identified as *Aspergillus flavus* by mass spectrometry MALDI-TOF and the strains were classified by MALDI-TOF MS into chemotypes based on their aflatoxins and cyclopiazonic acid production. According to the partial sequencing of ITS and CaM genes, a representative subset of 38 *A. flavus* strains was confirmed. Overall, 75 *A. flavus* strains (86%) were characterized as producers of aflatoxins. The co-occurrence of at least two mycotoxins (AF/ZEA, FUM/ZEA, and AF/ZEA/FUM) was detected for five of the *Z. mays* samples (63%). Considering the high mycological bioburden and mycotoxin contamination, maize marketed in the metropolitan region of Asunción constitutes a potential risk to food safety and public health and requires control measures.

## 1. Introduction

Due to its economic relevance in the food and feed industry, maize (*Zea mays* L.) is one of the most important grains in the world [1]. In Paraguay, ten native maize breeds have been adapted to the country’s environmental conditions [2,3]. *Zea mays* var. amylacea is the most distributed maize breed and the main ingredient of several traditional Paraguayan dishes [2,4,5]. In Guaraní, a pre-Columbian language spoken by the majority of Paraguayans, *Zea mays* var. amylacea is called *Avatí morotĩ*. In addition, *Zea mays* var. indurata (*Avatí locro* or *Tupi Morotĩ* in Guaraní) makes up a portion of the main staple food in the country [6,7].

In Paraguay, maize produced by family farming is primarily destined for human consumption. Usually, maize reaches the markets after local farmers sell their harvest to agro-distributors [8,9]. Maize grains are generally dried by sun exposure until reaching a moisture content of 12–14%. According to good agricultural practices (GAP), the grains should be stored under controlled temperature and humidity conditions [4,8,10,11]. However, these standards are not rigorously followed by local producers, which may lead to the spoilage fungi growth. During the production chain, maize is susceptible to both fungal infection and contamination with potential mycotoxin-producing fungi. Grain harvest and storage are usually the most critical stages for fungal growth and mycotoxin production [12,13].

Mycotoxins are low-molecular-weight secondary metabolites produced by fungi, mainly belonging to the genera *Aspergillus*, *Fusarium,* and *Penicillium* [14,15,16,17,18]. Strains of *Aspergillus* section *Flavi* are mostly responsible for producing the aflatoxins B1, B2, G1, and G2. Aflatoxin B1 (AFB1) is a naturally occurring fungal metabolite found in several crops worldwide (e.g., maize, dried foods, nuts, and so forth) [19]. AFB1 is known as being a hepatotoxic, teratogenic, carcinogenic, and immunotoxic compound to humans and animals [19,20]. Protracted exposures to AFB1 may trigger different acute and chronic effects on human health [17]. Furthermore, if ruminant animals consume maize-based feed contaminated with AFB1, this mycotoxin can be metabolized in the liver of the animal into aflatoxin M1 and then be excreted into its milk [21,22].

*Aspergillus* section *Flavi*, the greatest contaminant of grains in storage conditions, has a wide genetic intra-species variability, which makes accurate species identification difficult. The production of aflatoxins by *Aspergillus* section *Flavi* is multifactorial [19] and fungal identification based on a polyphasic approach is recommended [16,17].

Fungal polyphasic identification consists of the association of morphological, physiological, and molecular characteristics along with proteomic profiles of matrix-assisted laser desorption/ionization time-of-flight mass spectrometry (MALDI-TOF MS) [23,24,25,26,27]. Morphological identification is based on macro- and micro-morphological characteristics [28,29]. Physiological characterization includes the production of extrolites, which can be determined by chromatographic and/or spectrometric techniques [16,25]. Molecular biology techniques can be used to both identify fungal species and determine their mycotoxigenic potential; in this last case, this is based on the study of their mycotoxins’ biosynthesis pathways. Mass spectrometry by MALDI-TOF is a useful tool for both fungal species identification and mycotoxin detection on fungal colonies or directly on food products [16,27,29,30].

In order to predict risks and prevent mycotoxin contamination, an estimation of the fungal population in maize grains is valuable [12,31]. The environmental conditions of tropical and subtropical regions are optimal for fungal growth and mycotoxin contamination. In addition, developing countries located in tropical and subtropical regions often have low standards of agricultural and manufacturing practices, increasing the likelihood of fungal contamination [15,19].

The aim of the present study was to identify, based on a polyphasic approach, fungal strains belonging to *Aspergillus* section *Flavi* isolated from *Zea mays* var. indurata and *Z. mays* var. amylacea that are marketed in the metropolitan region of Asunción (Paraguay); another aim was to determine the fungal capability to produce mycotoxins.

## 2. Materials and Methods

### 2.1. Sampling

Samples of both *Z. mays* var. indurata (*avatí locro*) and *Z. mays* var. amylacea (*avatí-morotĩ*) were collected from January to March 2016. Samples of *Z. mays* var. indurata (*avatí locro*) were collected from 5 different sampling points (SP1 to SP5) in the metropolitan region of Asuncion, Paraguay: (SP1) Fernando de la Mora (25°19′36.06″ S, 57°34′20.65″ W); (SP2) Asunción (25°17′59.30″ S, 57°37′15.87″ W); (SP3) Limpio (25°10′40.00″ S, 57°29′2.36″ W); (SP4) Luque (25°16′14.86″ S, 57°29′16.43″ W); and (SP5) San Lorenzo (25°20′43.39″ S, 57°30′37.68″ W); meanwhile, samples of *Z. mays* var. amylacea (*avatí-morotĩ*) were collected from 3 different sampling points: (SP1) Fernando de la Mora; (SP2) Asunción; and (SP3) Limpio.

### 2.2. Composed Sample Preparation

For each sampling point, 20 kg each of *Z. mays* var. indurata (*avatí locro*) and *Z. mays* var. amylacea (*avatí-morotĩ*) were obtained from 4 different sellers (5 kg per seller). Final maize samples were obtained as a composed sample containing 5 kg each of *Z. mays* var. indurata (*avatí locro*) and *Z. mays* var. amylacea (*avatí-morotĩ*). After sample collection, the packages were tagged, refrigerated, transported to the Multidisciplinary Centre of Technological Research (CEMIT–UNA, Asunción, Paraguay), and stored at 4 °C. A total of 8 samples were obtained being distributed as 5 samples of *Z. mays* var. amylacea and 3 samples of *Z. mays* var. indurate.

### 2.3. Mycological Analysis of Z. mays var. indurata and Z. mays var. amylacea

For mycobiota isolation, 500 g of each maize-composed sample was disinfected by immersion in 3.0% sodium hypochlorite aqueous solution for 3 min. Then, 10 individual maize grains were randomly selected and plated on malt extract agar (MEA, malt extract 20 g L^−1^, mycological peptone 1 g L^−1^, agar 20 g L^−1^, glucose 20 g L^−1^). The analyses were performed with 5 replicates and were incubated for 7 days at 25.0 ± 2.0 °C.

Fungal colonies were selected based on their macro- and microscopic morphologies using taxonomic keys [32,33,34]. All colonies presenting macro- and microscopic characteristics of *Aspergillus* section *Flavi* were selected and subculture on MEA and incubated for 7 days at 25.0 ± 2.0 °C [28,29]. All isolates were preserved on 20% glycerol at −20.0 °C.

### 2.4. Morphological Identification of Aspergillus Section Flavi

For fungal morphological identification, spores of *Aspergillus* section *Flavi* colonies (*n* = 211) suspended in 1 mL of 0.2% agar were inoculated at 3 points on 90 mm diameter plates, each containing MEA and Czapek yeast extract agar medium (CYA, agar 15 g L^−1^, K_2_HPO_4_ 1 g L^−1^, sucrose 30 g L^−1^, yeast extract 5 g L^−1^, 10 mL of Czapek concentrate, 1 mL of trace metal solution). Each plate was incubated at 25 °C and then 37 °C on MEA and on CYA, respectively, in the dark, for 7 days.

Phenotypical identification was based on macro- and microscopic traits, with appropriate keys being used [28]. Macroscopic characteristics included diameter size, colony color, and texture and were recorded using a Labomed^®^ microscope coupled with a digital camera MDCE-5C USB 2.0 and the software Scope Image 9.0^®^ [28,29].

To analyze the sclerotia production, the 211 *Aspergillus* section *Flavi* strains previously identified by morphological traits were cultivated on CYA media and incubated at 30 °C for 10 days. After the 10-day period, the diameters of 30 randomly selected sclerotia were measured and classified into S strain (small <400 µm) and L strain (large ≥ 400 µm) groups [35]. All *Aspergillus* strains isolated in the present study were deposited at the Culture Collection of Microorganisms, Universidad Nacional de Asunción (CCM–CEMIT–UNA, Asunción, Paraguay).

### 2.5. MALDI-TOF MS Profiling

*Aspergillus* strains previously identified by classical morphology as *Aspergillus* section *Flavi* were analyzed by MALDI-TOF MS. For MALDI-TOF MS analysis, isolates were inoculated on 60 mm Petri dishes containing MEA medium and were incubated at 28 °C for 3 days. The fungal cells were transferred into 1.5-milliliter microtubes containing 500 μL of ethanol 70% (*v*/*v*, HPLC grade) and glass beads (Sigma-Aldrich, St. Louis, MO, USA). Microtubes were vortexed for 5 min and sonicated for 10 min, twice.

Each sample (1 μL) was transferred into the MALDI sample plate (Bruker Daltonics, Bremen, Germany) and, once dried, 1 μL of matrix solution was added [CHCA-alpha-cyano-4-hydroxycinnamic acid (Fluka, Buchs, Switzerland) saturated in a solution composed of 30% (*v*/*v*) acetonitrile, 69.9% (*v*/*v*) H_2_O, and 0.1% (*v*/*v*) trifluoroacetic acid]. After air-drying, mass spectra were obtained using the MALDI-TOF MS Autoflex Speed (Bruker Daltonics, Bremen, Germany), which was equipped with a smart beam laser source (355 nm). All samples were analyzed in triplicate.

The final spectra were generated by summing together the 20 laser shots accumulated per profile and the 60 profiles produced per sample, which led to a total of 1200 laser shots per spectrum without saturation in the range of *m*/*z* 2000–20,000 Da [36]. The spectra were analyzed using the MALDI Biotyper Compass 4.1 (Bruker Daltonics, Bremen, Germany) software and were compared with those spectra archived in the software library for filamentous fungi.

The results of the fungal identification were presented as logarithmic scores between 0.000 and 3.000. Scores above 1.700 were considered of high similarity. The dendrogram of spectral similarity was obtained by using an agglomerative clustering algorithm via the MALDI Biotyper Compass 4.1 software (Bruker Daltonics, Bremen, Germany) [36].

### 2.6. Molecular Identification of Aspergillus Section Flavi

For the molecular biology identification, fungal strains were grown in 250 mL of malt extract broth media under shaking conditions (150 rpm) for 24 h at 25 °C. For DNA extraction, 100 mg of mycelia from each isolate was filtered, weighed, and stored at −80 °C; DNA extraction was performed in liquid nitrogen using the lysis buffer CTAB (100 mM Tris–HCl pH 8.5; 1.4 M NaCl; 20 mM EDTA; 2% CTAB). The DNA was quantified using a spectrophotometer (DS-11-DeNovix) [37].

The polymerase chain reaction (PCR) amplifications were carried out using the primer sets ITS1 (TCCGTAGGTGAACCTGCGG), ITS4 (TCCTCCGCTTATTGATATGC) [29,38], CMD 5 (CCGAGTACAAGGARGCCTTC), and CMD6 (CCGATRGAGGTCATRACGTGG) [29,39].

PCR amplifications were performed in 25 µL of a reaction mixture containing a MgCl_2_-free reaction buffer, 1.5 mM of MgCl_2_, 1.25 U of Taq polymerase, 200 µM each of dNTP, 0.2 µM each of primer, and 1 ng/µL of template DNA. The PCR was carried out as follows: (1) 95 °C for 4 min; (2) 30 cycles of the following three steps: 1 min at 95 °C, 1 min at 55 °C, 2 min at 72 °C; and (3) a final 10 min at 72 °C [29]. PCR products were sent for sequencing at Macrogen Inc. (Seoul, Republic of Korea), where an automated PE Applied 169 Biosystems ABI-3730 sequencer was used.

The sequences obtained were base-called using the software seqtrace-0.9.0^®^ and were subsequently compared with public databases (GenBank database at the National Center for Biotechnology Information (NCBI) and Westerdijk Fungal Biodiversity Institute (WI, previously CBS-KNAW) with the BLAST algorithm. Sequences were aligned with the Clustal W program, with a penalization gap-opening setting of 15 and a gap extension of 6.66 for paired and multiple alignments, using the MEGA 7.0.26 software [40]. The sequences obtained were submitted to the National Center for Biotechnology Information (NCBI) database (Table S2).

### 2.7. Phylogenetic Analysis and Haplotype Network

The optimal substitution model was calculated using the Akaike information criterion (AIC) with MEGA 7.0.26 software [40]. The phylogenetic tree was prepared via the maximum likelihood method. All positions containing gaps and missing data were eliminated from the dataset (complete deletion option). Bootstrap values were calculated from 1000 replications of the bootstrap procedure using the MEGA 7 package.

In addition, a complementary phylogenetic analysis was performed by Bayesian inference using the Bayesian Markov chain Monte Carlo (MCMC) or Metropolis-coupled Markov chain Monte Carlo (MCMCMC) method implemented in the Bayes Phylogenies program (http://www.evolution.rdg.ac.uk/BayesPhy.html, accessed on 25 April 2022). The outgroup was *Aspergillus muricatus* NRRL 35674. The consensus tree was found by the majority rule consensus tree (50% threshold) using Geneious 9.0.5 software (https://www.geneious.com, accessed on 25 April 2022).

FigTree v.4.15.1 software (http://tree.bio.ed.ac.uk/software/figtree/, accessed on 3 September 2022) was used for the manipulation and graphical presentation of the resulting phylogenetic hypotheses. To evaluate the existence of a phylogeographic association between the sequences of the Paraguayan isolates and reference sequences from different geographic regions of the world [30], a haplotype network was elaborated with the ape, adegenet, pegas, and ggplot2 packages implemented in R version 3.6.1.

### 2.8. Molecular Characterization of Toxigenic Potential of Aspergillus flavus Isolates

A set of representative *Aspergillus* section *Flavi* strains identified by MALDI-TOF MS as *A. flavus* were selected for molecular biology analysis. The strains were assayed via multiplex PCRs for the detection of the structural genes *aflD*, *aflM*, and *aflP* and the regulatory gene *aflR*, using the primers described by Criseo et al. to do so [41]. To obtain positive/negative results regarding the aflatoxin biosynthetic genes, each DNA sample was tested in triplicate [41]. PCR products were analyzed by 1.5% agarose gel electrophoresis. The products were labeled with Diamond^TM^ nucleic acid dye and the band sizes were compared with 1 kb Plus and 100 bp molecular weight markers (Invitrogen^®^, Waltham, MA, USA). Gel electrophoresis visualization was performed using the Gel Doc^TM^_EZ Documentation System and Image Lab^TM^ 6.1 software, Bio-Rad^®^ (Hercules, CA, USA).

### 2.9. Aflatoxins and Cyclopiazonic Acid Detections by MALDI-TOF MS

To determine their mycotoxigenic ability, fungal strains were inoculated on 60 mm Petri dishes with CYA and yeast extract sucrose agar medium (YES, yeast extract 20 g L^−1^; sucrose 150 g L^−1^; agar 15 g L^−1^; MgSO_4_·7 H_2_O 0.5%; ZnSO_4_·7H_2_O 0.01%; CuSO_4_·5H_2_O 0.005%) and were incubated at 30 °C for 15 days.

For the mycotoxin extraction, three plugs of each sample were transferred into amber vials containing 1 mL of methanol-water (3:1, *v*/*v*). After 10 min of incubation at room temperature, samples were shaken at 150 rpm in a shaker for 30 min and centrifuged at 4500× g for 10 min. Supernatants were transferred into clean amber vials and the liquid phase was evaporated at room temperature overnight. The crude of each sample was re-suspended in 0.5 mL of methanol. Each sample (1 μL) was mixed on a paraffin film surface with 2 µL of CHCA solution [16,42] and 1 μL of the resulting suspension was transferred into the surface of a stainless steel MALDI sample plate (Bruker Daltonics, Bremen, Germany).

After air drying, mass spectra were obtained using the MALDI-TOF MS Autoflex Speed mentioned above. All samples were analyzed in duplicate. The spectral acquisition was acquired in the reflector positive mode, with an acceleration voltage of 19 kV under a mass range of *m*/*z* 200–400. Before the analysis, the instrument was externally calibrated using the Peptide Calibration Standard I (Bruker Daltonics, Bremen, Germany) [16,42].

The final spectra were generated by summing together the 20 laser shots accumulated per profile and the 60 profiles produced per sample, leading to a total of 1200 laser shots per summed spectrum. The MALDI Biotyper Compass 4.1 (Bruker Daltonics, Bremen, Germany) software was used to compare the obtained spectra with the spectra of the following standards: aflatoxin B1 (C_17_H_12_O_6_ = 312.280 Da), aflatoxin B2 (C_17_H_14_O_6_ = 314.289 Da), aflatoxin G1 (C_17_H_12_O_7_ = 328.273 Da), aflatoxin G2 (C_17_H_14_O_7_ = 330.29 Da), and cyclopiazonic acid (C_20_H_20_N_2_O_3_ = 336.391 Da) (Table S1) [16,42]. Peaks of *m*/*z* corresponding to protonated metabolites [M^+^H]^+^ or forming adducts with Na^+^ or K^+^ cations (cationization) were detected (Table S1 and Figure S3).

### 2.10. Mycotoxin Quantification in Maize

The mycotoxin content in the maize samples was determined by direct competitive enzyme-linked immunosorbent assays (ELISA). All samples picked from the same location were mixed according to the type of maize: *Z. mays* var. indurata (locro) or *Z. mays* var. amylacea (*avatí-morotĩ*). Mycotoxin content was analyzed as follows: total aflatoxins (AFs) [43], total fumonisins (FUMs) [44], total zearalenones (ZEAs) [45], and T-2 toxin (T-2) [46]. This was conducted using an ELISA kit AgraQuant^®^ from Romer Labs (Newark, DE, USA).

Each maize sample (20 g) was milled with a grain mill. An extraction of samples was made with a methanol:water (70:30 *v*/*v*) solution and the procedure was continued following the manufacturer’s recommendations. Optical densities (ODs) were determined using the microwell strip reader at 450 nm in a MultiSkan^TM^ FC (Thermo Scientific^®^, Waltham, MA, USA) system. The samples’ ODs were compared with the ODs of the standards and the obtained concentrations were determined in ppb (μg.kg^−1^).

### 2.11. Data Analysis

The mean fungal frequencies and the mean aflatoxin concentrations were subjected to a three-way ANOVA (multivariate) test, followed by Tukey’s HSD test (*p* < 0.05) using the GraphPad Prism 8.0.0. software for Windows, San Diego, CA, USA, www.graphpad.com. A cluster analysis was performed and a dendrogram of phenotypic relatedness was constructed using the IBM^®^ SPSS^®^ Statistics software for Windows 25.0.

To evaluate the association between aflatoxin production and the sclerotia morphotype, a logistic regression analysis was performed using the IBM^®^ SPSS^®^ Statistics software for Windows 25.0. A principal coordinate analysis (PCoA) was performed using GenAlEx^®^ 6.5 software [47]. *Aspergillus flavus* isolates were selected by a random sampling method based on morphologically and physiologically characterized isolates representing the phenotypes of *Aspergillus flavus* (section *Flavi*), *Aspergillus tamarii* (section *Flavi*), *A. awamorii* (section *Nigri*), and *Aspergillus* section *Aspergillus*.

## 3. Results

### 3.1. Mycobiota and Frequency of Aspergillus Section Flavi

A total of 521 fungal isolates were obtained from assessed maize samples. The predominant genera were *Aspergillus*, *Fusarium*, and *Penicillium*. After a preliminary identification based on morphological characters, 291 isolates were confirmed as belonging to the genus *Aspergillus*. A total of 211 out of 291 fungal strains were identified as *Aspergillus* section *Flavi*, the most predominant taxonomic group within the *Aspergillus* group (72%). Other species were distributed within *Aspergillus* section *Aspergillus* (21%), *Aspergillus* section *Nigri* (5%), and *Aspergillus* section *Circumdati* (2%).

The highest fungal abundance was observed for the *Z. mays* var. amylacea samples, which were contaminated with strains of *Aspergillus* spp., *Fusarium* spp., and *Penicillium* spp. In the samples of *Z. mays* var. indurata, *Aspergillus* spp. was the main contaminant while *Penicillium* spp. was isolated at low frequency. No strains of *Fusarium* spp. were isolated from *Z. mays* indurata samples.

The fungal abundance on each *Z. mays* variety was different among the local markets. A similar diversity of fungal genera was observed in *Z. mays* var. amylacea from SP2, SP4, and SP5. In SP1, *Z. mays* varieties were more contaminated with *Aspergillus* than samples from other sampling points were. In SP3, the frequency of *Fusarium* spp. in the *Z. mays* var. amylacea samples was significantly higher (ca. 45%) compared to that of other SPs (Table 1).

### 3.2. Morphological Identification of Aspergillus Section Flavi

All *Aspergillus* section *Flavi* isolates were characterized by macro- and micro-morphological observations (Figure S1). The following phenotypic characteristics were observed: yellow/green colonies, smooth to finely rough conidia, and predominantly biseriate conidiophores. According to the results of the morphological observations, the greatest percentage of the isolates presented bisseriate and radiate heads (75.2%).

The sclerotia production was evaluated for the 211 strains previously identified by their morphological traits as *Aspergillus* section *Flavi*. Among them, 70% (*n* = 147) produced L-type sclerotia (≥400 µm) and 13% (*n* = 28) produced S-type sclerotia (<400 µm). The other 17% of the isolates (*n* = 36) did not produce sclerotia.

Isolates classified as *A. tamarii* (section *Flavi*), presented brown colonies and rough conidia. Isolates classified as *A. awamori* (section *Nigri*), presented dark brown/black colonies and pale yellow reverse colonies. In CYA media, cultures presented black colonies with white edges; meanwhile, on MEA media, cultures were grayish. The conidia were lightly rough to rough. Isolates classified as morphotype *Aspergillus* section *Aspergillus*, showed no growth in the CYA at 37 °C and showed limited growth in both the CYA at 25 °C and the MEA. In terms of the micro-morphology, 60% of the isolates showed the presence of ascospores; the other 40% showed the anamorphic phase with uniseriate conidiophores and conidia.

**Figure 1:**
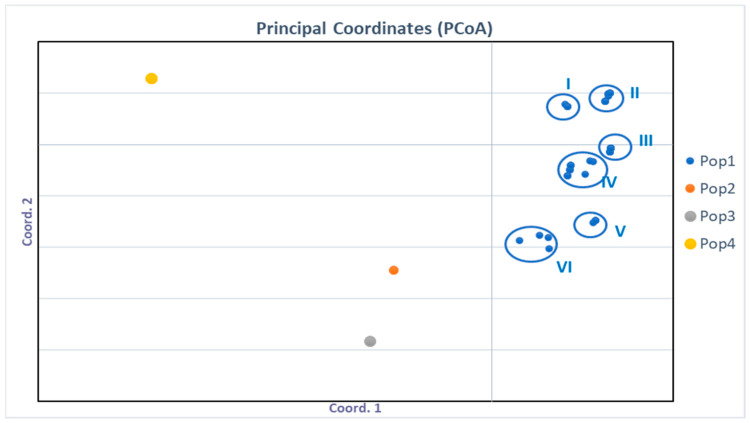
Principal coordinate analysis (PCoA) of *Aspergillus* population based on phenotypic features. Pop 1: Phenotype *A. flavus* (I–VI); Pop 2: Phenotype *A. awamorii*; Pop 3: Phenotype *A. tamarii*; Pop 4: Phenotype *Aspergillus* section *Aspergillus*.

Other *Aspergillus* strains, from species such as *Aspergillus tamarii* (section *Flavi*), *A. awamori* (section *Nigri*), and *Aspergillus* section *Aspergillus*, were used as outgroups in the principal coordinate analysis (PCoA) (Figure 1). According to the PcoA, based on the phenotypic characteristics, subgroups inside of the *A. flavus* population could be observed. These results indicated that the population of *A. flavus* isolated from maize was widely varied. According to the results presented herein, the population of *Aspergillus* section *Aspergillus* showed to be the most distant among the other populations (Figure 1).

### 3.3. MALDI-TOF MS Profiling

**Figure 2:**
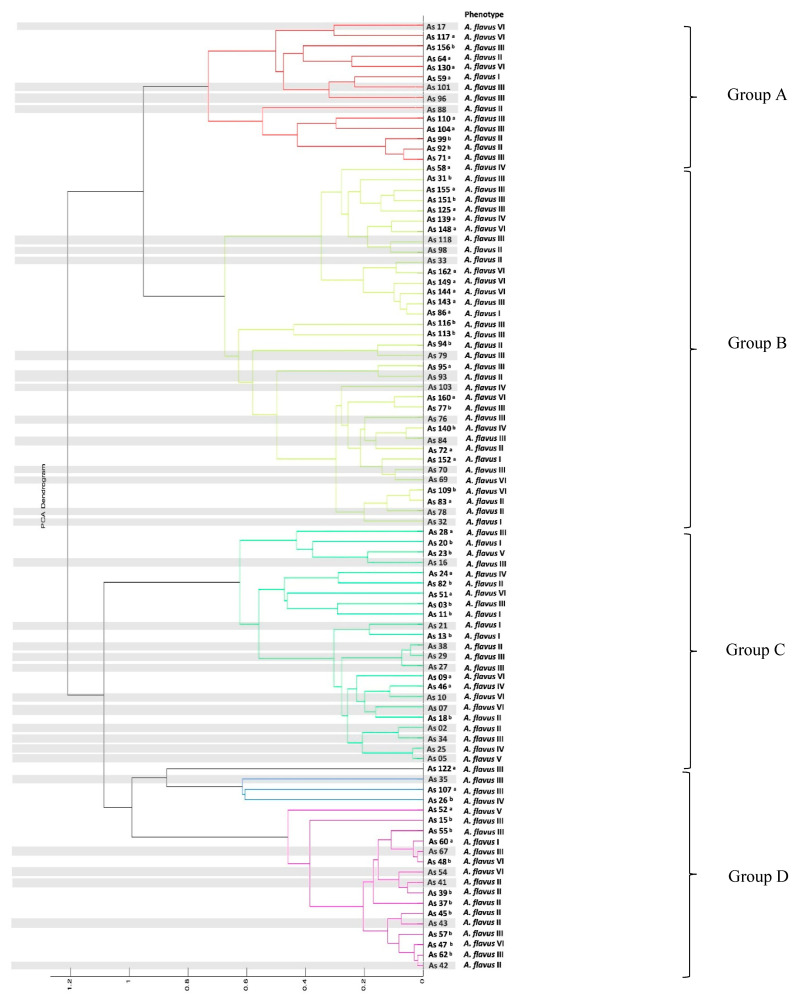
Dendrogram of proteomic profiles relatedness based on MALDI-TOF MS analysis for 92 *Aspergillus flavus* strains. Distances are measured as a percentage of spectral similarity. ^a^ MALDI-TOF ID only; ^b^ MALDI-TOF ID + Multiplex analyses; Gray box: MALDI-TOF ID + Molecular ID. The subtitle “Phenotype” indicates groups formed according to the PCoA.

Ninety-two *Aspergillus* section *Flavi* strains were identified, based on the proteomic profiles created by MALDI-TOF MS, as *A. flavus* (Figure 2). This corroborates the results obtained from the morphological analyses. In the MALDI-TOF MS dendrogram, four groups were observed (Groups A to D, shown in Figure 2). Based on the phenotypes obtained by PCoA analysis (Figure 1), no clear pattern was observed. However, in some groups, specific phenotypes predominate or share morpho-physiological characteristics (Groups A to D, shown in Figure 2).

In group A, 42% of the isolates corresponded to the phenotype *A. flavus* III; meanwhile, in group B, 40% of the isolates corresponded to the phenotype *A. flavus* III, 20% to phenotype II and another 20% to phenotype VI (Figure 2). In group C, the phenotypes observed were diverse; although, 42% of the isolates showed a uniseriate conidial head as a common morphological characteristic. In group D, 30% of the isolates corresponded to phenotype II; meanwhile, 40% of the isolates corresponded to phenotype III. However, the production of aflatoxin G was a common characteristic of all isolates of this clade (highlighted in purple in Figure 2).

### 3.4. Molecular Identification and Phylogenetic Analysis

A representative subset of 38 *Aspergillus flavus* isolates previously identified by MALDI-TOF MS was further identified based on the partial sequences of the nuclear ribosomal internal transcribed spacer (ITS) and calmodulin (CaM) genes. The phylogenetic trees were constructed based on the calmodulin (CaM or CMD5) gene sequences via two inference methods: maximum likelihood and Bayesian inference (Figure S2). Both analyses showed similar results.

### 3.5. Haplotype Network

**Figure 3:**
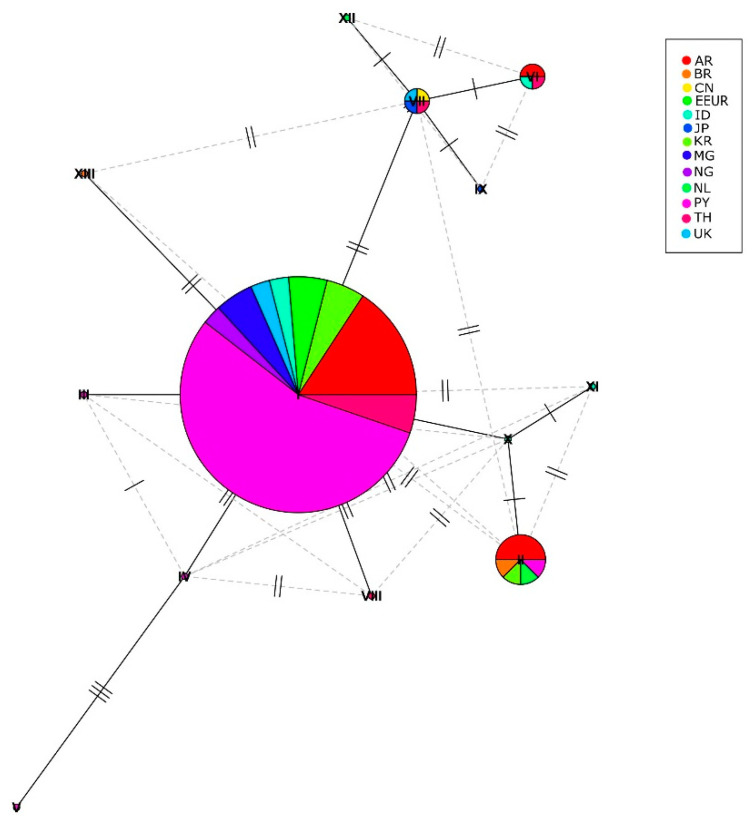
Haplotype network analysis based on partial CaM sequences (545 pb) of *A. flavus* isolates from different geographic origins; absolute frequency, *n* = 25. The circles and the Roman numerals indicate different haplotypes. The size of each circle is proportional to the frequency and each bar perpendicular to the solid line corresponds to a mutational step. Provenance, AR: Argentina, BR: Brazil, CN: China, EEUR: Eastern Europe, ID: Indonesia, KR: Republic of Korea, JP: Japan, MG: Madagascar, NG: Nigeria, NL: The Netherland, PY: Paraguay, TH: Thailand, UK: United Kingdom.

A haplotype network based on the CaM sequences (545 bp) was elaborated to evaluate the existence of a phylogeographic association among the *A. flavus* isolated from Paraguayan maize and the reference samples from different regions of the world (Table S2). Thirteen haplotypes were formed (Figure 3). Each circle in Figure 3 corresponds to a haplotype and their size is related to the frequency.

Most of the *A. flavus* isolates analyzed showed an association with those found in databases from other countries or regions, such as Argentina, Eastern Europe, Indonesia, Madagascar, Nigeria, Paraguay, Republic of Korea, Thailand, and the United Kingdom; these corresponded with haplotype I. Haplotype I is a dominant haplotype with a ubiquitous geographical distribution. For haplotype II, the isolates from Paraguay showed an association with samples from Argentina, Brazil, Eastern Europe, The Netherlands, and Republic of Korea. No mutational steps were observed, suggesting that they were a frequent haplotype. In addition, considering the distance and lower frequency, haplotypes IV and V (exclusively isolated from Paraguay), have the characteristics of new haplotypes.

### 3.6. Molecular Characterization of the Toxigenic Potential of Aspergillus flavus Isolates

**Figure 4:**
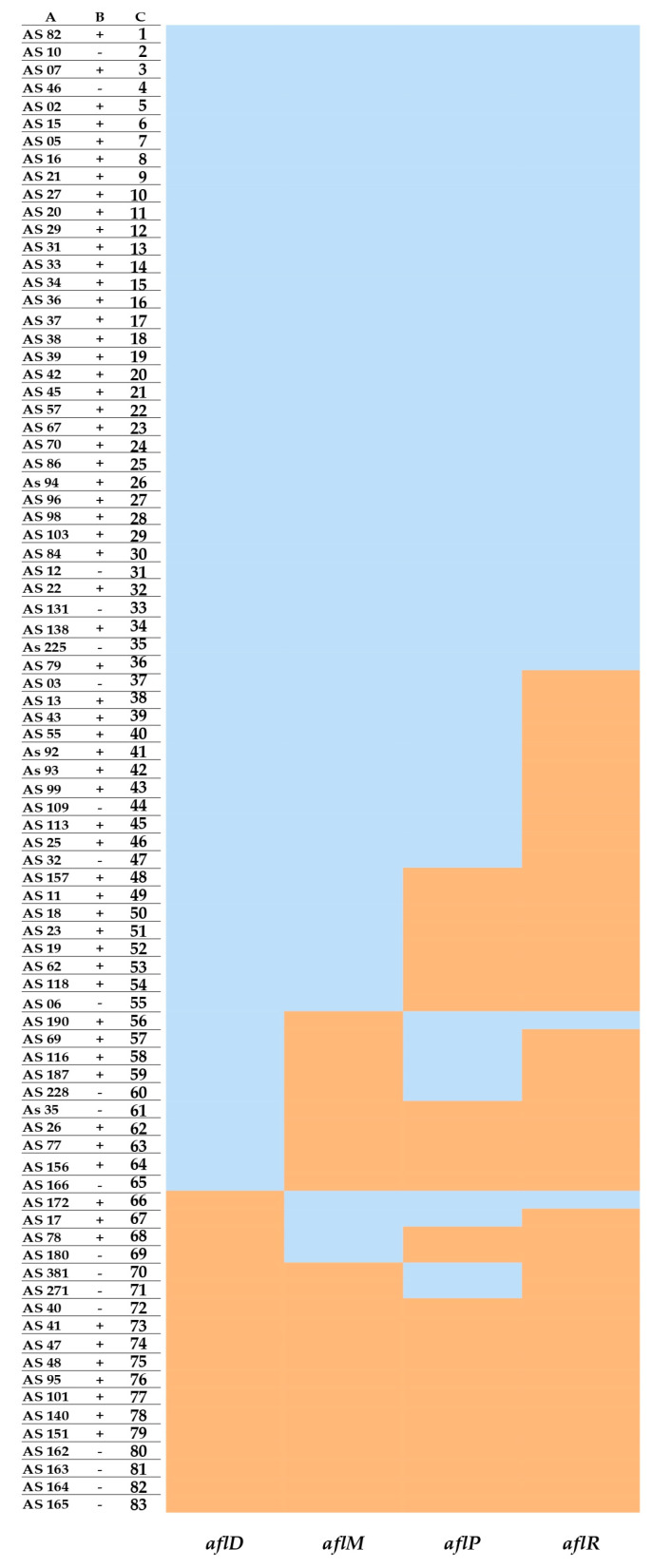
Multiplex heatmap for aflatoxin biosynthesis pathway genes (*AflD*; *aflM*; *aflP*; *aflR*) and aflatoxin production. Column A indicates the codes of *A. flavus* isolates; column B represents the presence (+) or absence (−) of aflatoxins, as determined by MALDI-TOF MS; and column C represents the code in the heatmap matrix. Orange indicates the absence (0) and light blue the presence (1) of the gene in the tested conditions.

The amplification patterns for 83 out of 92 strains of *A. flavus*, previously identified by MALDI-TOF MS, were evaluated. Only 36 out of 83 isolates presented bands corresponding to the four genes, *aflD*, *aflM*, *aflP*, and *aflR*. Data obtained showed that under tested conditions, 86% of fungal strains (*n* = 31) produced aflatoxins and 14% (*n* = 5) were not aflatoxin producers (Figure 4). There was no consistency when comparing the amplification patterns with the aflatoxin production, except in four isolates (AS 162, 163, 164, and 165), which did not amplify for the four genes and did not produce aflatoxins.

### 3.7. Aflatoxins and Cyclopiazonic Acid Detections by MALDI-TOF MS

All 92 *Aspergillus* strains were assayed by MALDI-TOF MS for aflatoxins and cyclopiazonic acid detection. A total of 86% of the isolates were aflatoxigenic while 4% of them presented mass peaks corresponding to cyclopiazonic acid (CPA) (Table 2 and Figure S3).

Based on the mass spectra obtained, the *A. flavus* isolates were grouped according to their chemotypes, as shown in Table 2. *Aspergillus flavus* isolates presented a variable number of peaks in their mass spectra, according to their ability to produce other kinds of metabolites.

The chemotypes VI (AFG) and VII (AFB + AFG) were predominant, being observed for 68% of the isolates evaluated. These were followed by chemotypes III (AFB) and V (non-aflatoxin producers), being associated with 19% and 14% of isolates, respectively. The remaining isolates were distributed between chemotypes II (AFB, AFG, CPA) and I (AFB, CPA) (Table 2). A unique fungal isolate (As 13) produced AFG and CPA and was named chemotype VIII. A correlation between the type of sclerotia and the probability of aflatoxin production was evaluated (*n* = 92); although, no statistical significance was observed (*p* > 0.05).

### 3.8. Mycotoxin Quantification in Maize

In Table 3, the concentrations of each total of the AFs, ZEAs and FUMs are presented as a mean of three replicates. In the present study, the concentration of total AF in *Z. mays* var. amylacea maize, as determined by ELISA, showed a contamination range spanning from 1.67 ± 0.2 to 20.75 ± 0.6 µg·kg^-1^. For the *Z. mays* var. indurata from SP1 and SP2, total AF concentrations were below the limit of detection (LOD < 1 µg.kg^−1^). Meanwhile, from SP3, the total AF concentration was observed as 2.30 ± 0.7 µg.kg^−1^ (Table 3).

For the T2 toxin, all samples analyzed showed values below the LOD (32 µg·kg^-1^). For ZEA, the obtained concentrations ranged from 21.64 ± 1.6 to 30.09 ± 3.0 µg.kg^−1^ in *Z. mays* var. amylacea and from 22.69 ± 2.0 to 247 ± 6.7 µg.kg^-^ in *Z. mays* var. indurata. Regarding FUM, all of the samples of *Z. mays* var. indurata analyzed showed a FUM concentration below the LOD (200 µg.kg^−1^). In *Z. mays* var. amylacea, the FUM concentrations ranged from 329.65 ± 36.8 to 1782.81 ± 271.0 µg.kg^−1^, except for the samples from SP4, which showed values below the LOD. The co-occurrence of at least two mycotoxins (AF/ZEA, FUM/ZEA, and AF/ZEA/FUM) was detected in 71% of the samples analyzed.

The ANOVA performed with the total AF concentrations, in terms of the samples of *Z. mays* var. amylacea from SP1, SP4, and SP5, differed (*p* < 0.01); there was variation among these three SPs. Considering only the results above the LOD, the *Z. mays* var. amylacea from SP1 showed the highest concentration of total AF; meanwhile, the lowest total AF concentrations were observed in samples from SP4. Samples from SP3 were excluded from the analysis because their concentrations were below the LOD.

## 4. Discussion

Paraguay displays a pool of *Z. mays* native germplasm. This agricultural product is intrinsically associated with the Paraguayan culture, being initially used as a nutritional source and raw material for the ceremonial drinks of native communities. Until now, maize remains an important staple food for the Paraguayan population. Despite this, the food safety of Paraguayan maize varieties has been poorly considered.

In the present study, an analysis of the mycological bioburden of *Z. mays* var. indurata and *Z. mays* var. amylacea was performed. The *Aspergillus* spp. and *Fusarium* spp. show the highest incidence in *Z. mays* var. amylacea. Conversely, in *Z. mays* var. indurata, only *Aspergillus* spp. was dominant.

Sepúlveda et al. [32] analyzed the *Aspergillus* population in maize from Argentina. The authors reported that *A. flavus* (*n* = 752) was the most abundant strain. Moreover, the authors reported significant isolation of *Fusarium* sp., followed at a lower frequency by the *Penicillium*, *Rhizopus*, and *Absidia* species [32]. Similarly, Gasperini et al. [48] reported that the *Fusarium*, *Penicillium,* and *Aspergillus glaucus* groups were the major contaminating genera in genetically modified (GM) and non-GM maize kernels samples from Brazil.

Regarding the maize variety analyzed in the present study, *Z. mays* var. indurata presented less fungi contamination. This possibly occurred due to the physical process that maize is subjected to before its commercialization. In this physical process, the pericarp is excluded, eliminating part of the field contamination. Furthermore, the *Z. mays* var. indurata is a flint-type corn with a hard endosperm, unlike *Z. mays* var. amylacea, which shows a very soft endosperm. The hard endosperm could provide a mechanical barrier to spoilage fungi damage [4,49].

Concerning the correct identification of food-contaminating fungi, in the present study, MALDI-TOF MS proved to be helpful in the identification of the *Aspergillus* section *Flavi* species. Quéro et al. [50] used MALDI-TOF MS to identify 68 isolates belonging to 23 species of *Aspergillus* section *Flavi*. The authors reported the correct identification of 99.65% of the fungal strains.

In the present study, 211 fungal strains were previously classified as *Aspergillus* section *Flavi* by morphological methods, which was not sufficient for species identification. Therefore, MALDI-TOF MS analysis was used as an additional tool. Herein, four clades were observed in the dendrogram obtained from the MALDI-TOF mass spectra results. These clades were not completely related to the phenotypic profile; consequently, it was not possible to differentiate aflatoxigenic isolates from non-aflatoxigenic isolates. These results are corroborated by previous studies in which the separation of aflatoxigenic isolates from non-aflatoxigenic isolates was not achieved by MALDI-TOF MS [51].

Similarly, Silva et al. 2015 [52] used the polyphasic approach to identify 35 *Aspergillus* section *Flavi* isolates obtained from a market in Lavras, Brazil. The MALDI-TOF MS analysis was not sufficient enough to differentiate aflatoxigenic from non-aflatoxigenic *Aspergillus*. However, the authors reported that in a polyphasic approach, the proteomic profile provided by MALDI-TOF MS, when associated with classical methodology, increased the reliability of the results.

Considering the above, MALDI-TOF mass spectrometry is a powerful tool to differentiate closely related fungal species. Additionally, a polyphasic approach is recommended for the identification of cryptic fungal species, such as *Aspergillus* section *Flavi* [30,50,51,52]. In order to increase the identification efficiency and accuracy at the fungal-species level, different studies suggested the importance of expanding the MALDI-TOF MS database of reference strains [53,54,55,56]. Such databases must include *Aspergillus* section *Flavi* autochthonous strains isolated from diverse substrates. Likewise, the MALDI-TOF mass spectra obtained in the present work can be used to supplement MALDI-TOF MS databases by adding isolates from maize of Paraguay, which has its own characteristics and genetic variability.

As mentioned above, for cryptic species, a comprehensive analysis including the results obtained from morphological, physiological, and molecular methods (polyphasic approach) was necessary [56,57]. The molecular identification of *Aspergillus* spp. is based on the amplification and partial sequencing of the ITS and CaM, or other genes, such as beta-tubulin and RPB2 (RNA polymerase II second largest subunit) [29,58,59].

Combining the information obtained from ITS and CaM sequencing is pivotal for the identification of the *Aspergillus* section *Flavi* isolates. In the present study, the identification of 38 *Aspergillus flavus* is supported by molecular biology information. The molecular biology data were used to support the previous identification based on morphology and MALDI-TOF MS analyses.

In addition, with the haplotype network analysis, the phylogenic data confirmed that most of the isolates were similar, in terms of the CaM gene, to *A. flavus* isolates from different geographical regions, such as Argentina, Eastern Europe, Indonesia, Nigeria, South Korea, Thailand, and the United Kingdom. However, three haplotypes (III, IV, and V) were more distant from the others, suggesting that these may be exclusively from Paraguay [60].

In addition to the identification achieved by the polyphasic approach, in the present work, an analysis of physiological characteristics was performed. Physiological characteristics are relevant for the comprehension of biodiversity populations of *A. flavus* in maize used for feeding humans and livestock.

MALDI-TOF mass spectrometry is a highly sensitive technique used to detect organic molecules present in low concentrations in different kinds of matrices. MALDI-TOF MS can be used to detect fungal secondary metabolites [61,62] and has previously been used to characterize *Fusarium* FUM-producers’ strains (16).

In the present study, 86% (*n* = 79) of the *A. flavus* strains were characterized by MALDI-TOF MS as producers of aflatoxins. The analyzed isolates were categorized into eight chemotypes based on the classification of Vaamonde et al. [63] and Razzaghi-Abyaneh et al. [64], with modifications included. Herein, 34 isolates (37%) were classified as chemotype VI and 24 isolates (26%) as chemotype VII; both were producers of AFG and together represented a total frequency of 63% of the whole assessed group of *A. flavus* strains.

Similarly, Camiletti et al. [65] studied the toxigenic potential of *Aspergillus* section *Flavi* strains isolated from corn ears. The authors reported that 79% of the *Aspergillus* section *Flavi* isolates produced AFB and AFG and a total of 95% showed toxigenic potential.

Probst et al. [66] evaluated the aflatoxin production of *A. flavus* strains using in vitro and in vivo techniques. According to the authors, the AFB- and AFG-producers’ strains are more toxigenic and, therefore, produce higher aflatoxin concentrations. Other than that, two isolates were classified as chemotype II and one was classified as chemotype I, with both belonging to the CPA-producing chemotypes.

Cyclopiazonic acid is the main contaminant of Kodo millet grain (*Paspalum scrobiculatum*), causing immunotoxic and cytotoxic effects in end consumers [67,68]. The strains that produced AFG and CPA were included in chemotype VIII in the present study.

The data presented here showed that the frequency of CPA-producing strains was lower than what was shown in the results found by Vaamonde et al. [63]. According to Vaamonde et al. [63], 54% of *A. flavus* strains isolated from peanuts were classified as chemotype I (AFB+, CPA+); but, in wheat and soybean, this was less frequent (13% and 5%, respectively) [63]. Other than that, 14 strains were classified as chemotype V, non-aflatoxigenic strains.

According to Olarte et al. [69], the cluster of genes involved in aflatoxin production is affected during sexual reproduction and this is not only in heterokaryosis, explaining the wide diversity of the *A. flavus* population [69]. The search for non-aflatoxigenic strains of *A. flavus* has increased since exclusion by competition has been shown to be an efficient way of reducing aflatoxin levels in maize [70,71,72]. Some of the requirements for these organisms to be used in the field are that they should be native strains, non-cyclopiazonic acid (CPA) producers, and genetically stable [73,74].

Another well-studied characteristic of the *A. flavus* population is the sclerotia. In the present study, 83% of *A. flavus* isolates produced sclerotia at 30 °C, a common room temperature in Paraguay (average of 31.5–34.9 °C to 20.1–23.7 °C) [75]. This points out that *A. flavus* has a great potential to spread and survive in this agro-ecosystem [32,64].

The predominant sclerotia among the analyzed *A. flavus* strains was L-type (70%), >400µm. Similarly, Rocha et al. [76] reported a frequency of 66% of *A. flavus* being isolated from maize samples (*n* = 200) from different geographical regions and climate conditions in Brazil. All isolates were classified as sclerotia L-type [76]. Likewise, Perrone et al. [77] analyzed 91 samples of freshly harvested maize from Nigeria and Ghana. The *A. flavus* were isolated in high frequency (98.5%) and all of them were sclerotia L-type. Probst et al. [78] evaluated 364 maize grain samples from 18 African countries and determined that 84.2% of the fungi species isolated were *A. flavus* type L.

Nesci and Etchevery [79] isolated and characterized *Aspergillus* species from soil, stubble, and insect samples from maize fields in three periods: pre-sowing, in-crop, and post-harvest. The frequency of *A. flavus* sclerotia producers ranged from 18 to 34%. However, all of the isolates that produced sclerotia were L-type.

According to Bayman and Cotty [80], due to the greater production of conidia, the morphology of L-type strains favors fungi colonization, especially when compared to S strains. Mauro et al. [81] isolated 138 *A. flavus* strains, all L-type, from maize samples from five districts in Northern Italy. According to the authors, *A. flavus* type L is the causal agent of aflatoxin contamination in maize in Italy [81]. Similarly, Astoreca et al. [82] reported that L-type strains were more toxigenic than S-type strains. Other studies suggested an association of the L morphology with the antioxygenic potential [64,83,84,85].

The present study is the first report on S- and L-type *A. flavus* strains isolated in Paraguay that produce both aflatoxins B and G. Until now, no correlation between sclerotia morphology and AF biosynthesis could be determined. Different studies have pointed out that AF production is related to multifactorial factors, such as genotypes, climate changes, and agricultural practices [19,81,85,86,87].

In the present study, PCoA grouped *A. flavus* isolates with different morphological characteristics, such as colony color, sclerotia type (e.g., L, S), and physiological markers (e.g., aflatoxin production), forming different phenotypic groups. The data presented herein corroborate the findings of previous studies [77,88], which point out that the *A. flavus* population is phenotypically diverse.

Regarding the mycotoxigenic characterization conducted by using molecular biology, 31 *A. flavus* isolates were amplified for AF genes (e.g., *aflD*, *aflM*, *aflP*, and *aflR*) and produced AFs. Conversely, the AF biosynthesis was not detected in five *A. flavus* strains; although, it amplified the four AF genes.

Gallo et al. [89] analyzed the presence of seven aflatoxin biosynthesis pathway genes, *aflD*, *aflM*, *aflO, aflP*, *aflQ*, *aflR*, and *aflS*, from *A. flavus* maize isolates from Italy using a PCR and determined four amplification patterns. They reported the presence of isolates that amplified all seven genes but did not produce aflatoxins. Oloo et al. [90] analyzed the AF biosynthesis genes’ profiles and their relationship with AF production in *A. flavus* isolates (*n* = 72). Among the seven AF biosynthesis genes assessed (*aflD*, *aflM*, *aflO*, *aflP*, *aflQ*, *aflR*, and *aflS*), only the presence of *aflD* and *aflS* significantly increased the amount of total aflatoxin production.

Based on the literature, the population of aflatoxin-producing and non-producing *A. flavus* is genetically diverse. Nevertheless, works such as the present study are useful for determining the strains responsible for contaminating foods and characterizing isolates that can be used in terms of biological control [74,76,91].

The presence of mycotoxin-producer fungi in maize is considered a primary indicator of mycotoxin contamination. This correlation was confirmed in the present study. The samples of *Z. mays* var. amylacea detected with high contamination levels of *Aspergillus* and *Fusarium* strains also showed high concentrations of AF, FUM, and ZEA.

Regarding *Z. mays* var. indurate, ZEA was the most prevalent mycotoxin. The detection of ZEA, despite the absence of *Fusarium* strains in *Z. mays* var. indurata, suggests that fungal infection, as well as mycotoxin contamination, occurs mainly in the field [13,15]. It has been reported that *Fusarium* sp. can also be present in stored grains, even though over time, this is shifted by the interaction with the mycobiota and by the decrease in relative humidity [34,92].

The European Commission (EC) established rigorous legislation for mycotoxins in maize products, considering maize’s different processing and derivative products. For total AFs (AFB1 + AFB2 + AFG1 + AFG2) and for AFB1 in all cereals and products derived from cereals, including maize products, the EC has set the maximum allowed levels (MAL) at 4 µg.kg^−1^ and 2.0 µg.kg^−1^, respectively [93].

In addition, the EC has set the MAL of ZEA in maize, for direct human consumption, maize-based snacks, and maize-based breakfast cereals, at 100 µg.kg^−1^. For fumonisins (sum of FB1 and FB2), the MAL has been set at 1000 µg.kg^−1^. The base standard for MERCOSUR countries regarding AFs in maize is the technical regulation on maximum limits of aflatoxins MERCOSUR/GMC/RES N°. 56/94 [94]. The MERCOSUR regulation allows the maximum levels of total AFs (20 µg.kg^−1^) in maize grain and its derivatives to be five times higher than those set by the EC. In addition, no MAL was established for the *Fusarium* toxins (FB1, FB2, and ZEA) in maize products [94]. However, this study showed the presence of these toxins in the maize grains analyzed, pointing out the risks for the final consumer and demanding attention from the authorities responsible for decision-making in the food safety area.

In the present study, two samples of *Z. mays* var. amylacea from SP1 (20.75 ± 0.6 µg.kg^1^) and SP5 (6.65 ± 1.5 µg.kg^−1^) presented a total AF concentration higher than the limits established by the EC. Additionally, a sample of *Z. mays* var. amylacea from SP1 also exceeded the MAL of FUM (1782.81 ± 271.0 µg.kg^−1^).

For *Z. mays* var. indurate, one sample (247 ± 6.7 µg.kg^−1^) exceeded the MAL for ZEA. Zearalenone is not carcinogenic but it has a potent estrogenic effect in animals, especially in pigs [95]. In humans, depending on the dose and exposure time, ZEA can cause several reproductive disorders, such as hyperestrogenic syndromes.

Due to the popular belief that it increases milk production, the intake of maize-based foods is higher among Paraguayan pregnant and puerperal individuals. In addition, maize-based foods are also part of the schools’ snack menus in Paraguay [96].

Previous studies have reported mycotoxin co-contamination in maize samples worldwide [97,98,99]. However, to the best of our knowledge, this is the first report of mycotoxin co-occurrence (AFs/ZEA/FUM/and AFs/ZEA) in maize samples from Paraguay. The combined intake of different types of mycotoxins may lead to a synergistic, or at least additive, effect that causes a risk to human health [17,100,101,102].

The present study contributes to the understanding of the biodiversity of the *A. flavus* population and the co-occurrence of aflatoxins, fumonisins, and zearalenone in the native Paraguayan maize commercialized in the local markets. In such local markets of Paraguay, maize is stored in improper conditions, piled up in burlap bags with no control over the temperature and humidity. The inadequate storage of maize can promote the growth of *Aspergillus*, *Fusarium,* and *Penicillium* strains, which grow in a wide range of temperatures and water activities (a_w_) [98,99,103,104,105].

To mitigate spoilage fungi and mycotoxin contamination in Paraguayan maize products, a combined strategy should be applied; as management strategies, the use of a tolerant germplasm, good agronomic practices, and good manufacturing practices could be included. Other than that, mycobiota characterization is a key element that should be used to mitigate mycotoxin contamination. Regarding this, the analysis presented herein is focused on the *Aspergillus* section *Flavi* characterization since this section includes the main aflatoxin producers [31,37,52,106,107,108,109,110,111,112,113].

## 5. Conclusions

This study provides the first comprehensive dataset regarding the genetic diversity and aflatoxigenic potential of *Aspergillus* section *Flavi* strains from *Zea mays* var. amylacea and *Zea mays* var. indurata, two maize ecotypes from Paraguay. Species of *Aspergillus* and *Fusarium* showed the highest incidence in *Z. mays* var. amylacea. In *Z. mays* var. indurata, species of *Aspergillus* were dominant.

Regarding mycotoxin contamination, AF, FUM, and ZEA were detected in one sample of SP1 and one sample of SP5 in *Z. mays* var. amylacea. Meanwhile, in *Z. mays* var. indurata, ZEA was detected in all samples. Despite the absence of the *Fusarium* strains, the detection of ZEA suggests that the field is a critical point for fungal infection and mycotoxin contamination during *Z. mays* var. indurata production. The detection of AF, FUM, and ZEA and the high mycological burden emphasizes the need for improvements regarding the *Zea mays* production chain.

In the present work, the fungi identification conducted using a polyphasic approach (morphological, physiological, MALDI-TOF MS profiles, and molecular biology) proved to be efficient for identifying and discriminating closely related species encompassed in *Aspergillus* section *Flavi*. More studies covering different agro-ecological zones and seasons are needed for better comparisons to be made among the mycotoxigenic mycobiota of Paraguayan *Zea mays*.

## Figures and Tables

**Table 1:** Frequency (Mean ± SD) of fungi isolated from the maize samples *Z. mays* var. indurata and *Z. mays* var. amylacea.

Maize	Sampling Points (SP)	Frequency (%)			
		*Aspergillus* spp.	*Fusarium* spp.	*Penicillium* spp.	Sterile Mycelia
*Z. mays* var. amylacea	SP1	100 ± 0 ^c^	4± 5 ^a^	0 ± 0 ^a^	0 ± 0 ^a^
	SP2	60 ± 5 ^b^	16 ± 6 ^b^	10 ± 3 ^b^	2 ± 1 ^ab^
	SP3	20 ± 13 ^d^	45 ± 6 ^d^	0 ± 0 ^a^	5 ± 7 ^ab^
	SP4	58 ± 9 ^b^	28 ± 8 ^c^	3 ± 4 ^a^	2 ± 2 ^ab^
	SP5	60 ± 4 ^b^	23 ± 8 ^bc^	3 ± 3 ^a^	9 ± 5 ^b^
*Z. mays* var. indurata	SP1	100 ± 0 ^c^	0 ± 0 ^a^	0 ± 0 ^a^	0 ± 0 ^a^
	SP2	11 ± 8 ^a^	0 ± 0 ^a^	0 ± 0 ^a^	4 ± 5 ^ab^
	SP3	22 ± 19 ^a^	0 ± 0 ^a^	1 ± 1 ^a^	0 ± 0 ^a^

Sampling Points: (SP1) Fernando de la Mora; (SP2) Asunción; (SP3) Limpio; (SP4) Luque; (SP5) San Lorenzo. Frequencies were compared by columnwithin species. Means with a common letter are not significantly different (ANOVA–Tukey’s Test, *p* < 0.05).

**Table 2:** Chemotypes, mycotoxins, mycotoxigenic strains frequency, and sclerotia production for *Aspergillus flavus* isolates.

Chemotype	Mycotoxins	N° Isolates (Frequency %)	Producers of Sclerotia (Frequency %)
I	AFB, CPA	1 (1%)	1/1 (100%)
II	AFB, AFG, CPA	2 (2%)	0
III	AFB	17 (19%)	16/17 (94%)
IV	CPA	0	0
V	Non-producer	13 (14%)	12/13 (92%)
VI	AFG	34 (37%)	25/34 (73%)
VII	AFB, AFG	24 (26%)	21/24 (87%)
VIII	AFG, CPA	1 (1%)	0

AFB: Aflatoxin B; AFG: Aflatoxin G; CPA: Cyclopiazonic Acid.

**Table 3:** Means of mycotoxin levels (µg.kg^−1^) from maize samples by ELISA.

Maize	Sampling Points (SPs)	Mycotoxins Level (µg.kg^−1^) ± SD			
		Total Aflatoxin ^a^	T2 ^b^	ZEA ^c^	FUM ^d^
	SP1	20.75 ± 0.6 *^A^	<LOD	24.07 ± 0.9 ^A^	1782.81 ± 271.0 ^#A^
	SP2	<LOD	<LOD	< LOD	<LOD
*Z. mays* var.	SP3	<LOD	<LOD	25.22 ± 3.0 ^A^	347.98 ± 58.1 ^B^
amylacea	SP4	1.67 ± 0.2 ^CD^	<LOD	30.09 ± 3.0 ^AB^	<LOD
	SP5	6.65 ± 1.5 ^B^	<LOD	21.64 ± 1.6 ^A^	329.65 ± 36.8 ^B^
*Z. mays* var.	SP1	<LOD	<LOD	22.69 ± 2.0 ^A^	<LOD
indurata	SP2	<LOD	<LOD	34.88 ± 1.9 ^B^	<LOD
	SP3	2.30 ± 0.7 ^D^	<LOD	247 ± 6.7 ^#C^	<LOD

^a^ LOD-AF: 1 µg.kg^−1^, ^b^ LOD-T2: 32 µg.kg^−1^, ^c^ LOD-ZEA: 20 µg.kg^−1^, ^d^ LOD-FUM: 200 µg.kg^−1^. * Southern Common Market (MERCOSUR) maximum allowable limit of total aflatoxins: 20 µg.kg^−1^. ^#^ Overcomes the European Food Safety Authority (EFSA) and Brazilian Health Regulatory Agency (ANVISA) maximum allowed levels. The upper-case letters indicate differences in mycotoxin concentrations. Means with a common letter are not significantly different (ANOVA, Tukey’s test, *p* > 0.05).

## Data Availability

Not applicable.

## Supplementary Materials

The following supporting information can be downloaded at: https://www.mdpi.com/article/10.3390/microorganisms11081879/s1. Table S1: MALDI-TOF mass spectra information (*m*/*z*) analyzed for secondary metabolite. Table S2: The *Aspergillus* sp. Isolates used in this study for molecular analyses. Figure S1: Morphological characteristics (macro- and microscopic) of *Aspergillus* morphotypes. (a) *A. flavus* section *Flavi*; (b) morphotype *A. awamori* (section *Nigri*): (c) morphotype *A. tamarii* (section *Flavi*); (d) morphotype *Aspergillus* section *Aspergillus*. First row: growth in CYA medium at 25 °C, second row: growth in CYA at 37 °C, third row: growth in MEA at 25 °C, fourth row: light microscope image at 40X magnification. Figure S2: Phylogenetic tree inferred from the partial CaM sequences showing the relationships among members of *Aspergillus flavus*. On the left: phylogenetic tree using the Bayesian inference based on the Bayesian Markov chain Monte Carlo (MCMC) or Metropolis-coupled Markov chain Monte Carlo (MCMCMC) model. On the right: maximum likelihood (ML) tree. ML bootstrap support values over 75%, and Bayesian posterior predictive values over 75% are shown above the branches. The tree is rooted with *Aspergillus muricatus* EF661434.1. Figure S3: MALDI-TOF MS spectra with standards for aflatoxins B1 (a), B2 (b), G1 (c), G2 (d); Cyclopiazonic Acid € and samples positive for AFB2, AS 36 (f) and negative AS 10 (g).
