# Supplementary material for: Species Identification and Mycotoxigenic Potential of Aspergillus Section Flavi Isolated from Maize Marketed in the Metropolitan Region of Asunción, Paraguay
Source: Microorganisms. 2023 Jul 25;11(8):1879. doi: 10.3390/microorganisms11081879 (PMC10458825; doi:10.3390/microorganisms11081879)
Supplement: Supplementary file 1 [file microorganisms-11-01879-s001.zip › microorganisms-2486865-supplementary.pdf]

Supplementary Materials:

Table S1: MALDI-TOF mass spectra information (m/z) obtained for secondary metabolites.

Compounds (m/z)	AFB1 (m/z)	AFB2 (m/z)	AFG1 (m/z)	AFG2 (m/z)	CPA (m/z)
[M+Na]+	335,267	337,27	351,263	353,278	359,374
[M+K]+	351,375	353,378	367,371	369,386	375,482
[M+H]+	313,285	315,288	329,281	331,296	337,392

AFB1: Aflatoxin B1; AFB2: Aflatoxin B2; AFG1: Aflatoxin G1; AFG2: Aflatoxin G2; CPA: Cyclopiazonic acid

Table S2: *Aspergillus* sp. isolates used in this study for molecular analyses.

Species	Code	Geographic Code/Synonym	Origin	GenBank Accession number		Reference
				ITS	CaM	
Aspergillus aflatoxiformans	CBS 143679	A. aflatoxiformans_Min_Nig	Nigeria	MG662388	MG518076	[30]
	CBS 135587	A. aflatoxiformans_Vwrang_Nig	Nigeria	MG662387	MG518077	[30]
Aspergillus alliaceus	DTO 363-F1	A. alliaceus_Esp	Spain	MH279432	MG518146	[30]
	DTO 363-E9	A.alliaceus_Pir_Esl	Slovenia	MH279431	MG518147	[30]
Aspergillus arachidicola	DTO 228-H9	A. arachidicola_Buf_AfrSur	South Africa	MG662384	MG518091	[30]
Aspergillus aspearensis	CBS 143672T	A. aspearensis_AspIsl_Ira	Iran	MG662398	MG518040	[30]
Aspergillus austwickii	CBS 143677T	A. austwickii_Abe_Nig	Nigeria	MG662391	MG518072	[30]
Aspergillus caelatus	CBS 763.97T	A. caelatus_EUA	USA	AF004930	MG518018	[30]
Aspergillus caelatus	DTO 276-I2	A.caelatus_Cor_Arg	Argentina	–	MG518108	[30]
Aspergillus cerealis	CBS 143674T	A. cerealis_Sha_Nig_1	Nigeria	MG662394	MG518063	[30]
	DTO 228-F1 = IBT 32070	A. cerealis_Sha_Nig_2	Nigeria	MG662392	MG518066	[30]
Aspergillus flavus	CBS 100927^{T} = NRRL 1957	A. flavus_Isl_Pac	South Pacific Islands	AF027863	EF661508	[30]
Aspergillus flavus	CBS 117637	A. flavus_For_Arg	Argentina	–	MG518010	[30]
Aspergillus flavus	CBS 11055 = ATCC 12073	A. flavus_Bra1	Brazil	FJ491463	MG518005	[30]
Aspergillus flavus	CBS 118.62	A. flavus_Bra2	Brazil	–	MG517996	[30]
Aspergillus flavus	CBS 119368	A. flavus_Chu_CorSur	South Korea	–	MG518002	[30]
Aspergillus flavus	CBS 120.51	A. flavus_Lon_Ing	United Kingdom	EF661549	MG518012	[30]
Aspergillus flavus	CBS 143688	A. flavus_CorSur1	South Korea	–	MG518144	[30]
Aspergillus flavus	CBS 143689	A. flavus_CorSur2	South Korea	–	MG518145	[30]
Aspergillus flavus	CBS 501.65	A. flavus_Ing	England	EF661563	MG518015	[30]
Aspergillus flavus	CBS 542.69	A. flavus_Kan_Jap	Japan	EF661554	MG518016	[30]
Aspergillus flavus	DTO 016-I5	A. flavus_Chi	China	–	MG518003	[30]
Aspergillus flavus	DTO 062-C7	A. flavus_Ind1	Indonesia	–	MG517985	[30]
Aspergillus flavus	DTO 062-C8	A. flavus_Ind2	Indonesia	–	MG517986	[30]
Aspergillus flavus	DTO 062-H7	A. flavus_Ind3	Indonesia	–	MG517987	[30]

Aspergillus flavus	DTO 066-C3	A. flavus_Ind4	Indonesia	–	MG517989	[30]
Aspergillus flavus	DTO 087-A3	A. flavus_Ifa_Mad1	Madagascar	–	MG517991	[30]
Aspergillus flavus	DTO 087-A4	A. flavus_Ifa_Mad2	Madagascar	–	MG517992	[30]
Aspergillus flavus	DTO 215-E5	A. flavus_Nig	Nigeria	–	MG518050	[30]
Aspergillus flavus	DTO 258-C9	A. flavus_Eur1	East. Europe	–	MG518095	[30]
Aspergillus flavus	DTO 258-D6	A. flavus_Eur2	East. Europe	–	MG518098	[30]
Aspergillus flavus	CBS 117638	A. flavus_Cor_Arg1	Argentina	–	MG518011	[30]
Aspergillus flavus	DTO 276-H7	A. flavus_Cor_Arg2	Argentina	–	MG518104	[30]
Aspergillus flavus	DTO 276-H8	A. flavus_Cor_Arg3	Argentina	–	MG518105	[30]
Aspergillus flavus	DTO 276-H9	A. flavus_Cor_Arg4	Argentina	–	MG518106	[30]
Aspergillus flavus	DTO 276-I1	A. flavus_Cor_Arg5	Argentina	–	MG518107	[30]
Aspergillus flavus	DTO 276-I3	A. flavus_Cor_Arg6	Argentina	–	MG518109	[30]
Aspergillus flavus	DTO 276-I4	A. flavus_Cor_Arg7	Argentina	–	MG518110	[30]
Aspergillus flavus	DTO 276-I5	A. flavus_Cor_Arg8	Argentina	–	MG518111	[30]
Aspergillus flavus	DTO 276-I6	A. flavus_Cor_Arg9	Argentina	–	MG518112	[30]
Aspergillus flavus	DTO 276-I7	A. flavus_Cor_Arg10	Argentina	–	MG518113	[30]
Aspergillus flavus	DTO 276-I8	A. flavus_Cor_Arg11	Argentina	–	MG518114	[30]
Aspergillus flavus	DTO 281-E2	A. flavus_Tai1	Thailand	–	MG518115	[30]
Aspergillus flavus	DTO 281-H8	A. flavus_Tai2	Thailand	–	MG518116	[30]
Aspergillus flavus	DTO 285-F6	A. flavus_Tai3	Thailand	–	MG518118	[30]
Aspergillus flavus	DTO 285-G3	A. flavus_Tai4	Thailand	–	MG518119	[30]
Aspergillus flavus	DTO 285-I4	A. flavus_Tai5	Thailand	–	MG518123	[30]
Aspergillus flavus	DTO 300-C7	A. flavus_Imp_Hol1	Netherlands	–	MG518124	[30]
Aspergillus flavus	DTO 300-D7	A. flavus_Imp_Hol2	Netherlands	–	MG518125	[30]
Aspergillus flavus	DTO 359-D7	A. flavus_CorSur1	South Korea	–	–	[30]
Aspergillus flavus	DTO 359-E1	A. flavus_CorSur2	South Korea	–	–	[30]
Aspergillus flavus	CBS 485.65 = ATCC 16870	A. flavus_Jap1	Japan	EF661563	MG518014	[30]
Aspergillus lanosus	CBS 650.74T	A. lanosus_Utt_Ind	Indonesia	FJ491471	MG518017	[30]
Aspergillus leporis	CBS 151.66T = ATCC 16490	A. leporis_Wyo_EUA1	USA	MH279391	MG518033	[30]
Aspergillus leporis	CBS 132153 = DTO 210-E1	A. leporis_Wyo_EUA2	USA	MH279396	MG518048	[30]

Aspergillus luteovirescens	CBS 620.95T	A. luteovirescens_Amz_Bra	Brazil	MG662406	MG51799 8	[30]
Aspergillus minisclerotigenes	CBS 117635T	A. minisclerotigenes_Cor_Arg	Argentina	EF409239	MG51800 9	[30]
	CBS 117633 = DTO 009-F5	A. minisclerotigenes_For_Arg	Argentina	MG662408	MG51800 7	[30]
Aspergillus mottae	CBS 130016T = DTO 223- C8	A. mottae_Brg_Por	Portugal	JF412767	MG51805 8	[30]
Aspergillus neoalliaceus	CBS 143681T = DTO 326-D3	A. neoalliaceus_RepChe1	Czech Republic	MH279420	MG51813 3	[30]
	DTO 326-E7	A. neoalliaceus_RepChe2	Czech Republic	MH279428	MG51814 2	[30]
Aspergillus nomius	CBS 260.88T= NRRL 13137	A.nomius_EUA	USA	AF027860	EF661531	[30]
	CBS 399.93 = DTO 301-I8	A. nomius_Zha_Chi	China	FJ491472	MG51812 7	[30]
Aspergillus novoparasiticus	CBS 126849T = DTO 223-C3	A.novoparasiticus_SP_Bra1	Brazil	MG662397	MG51805 5	[30]
	CBS 126850 = DTO 223- C5	A.novoparasiticus_SP_Bra2	Brazil	MH279415	MG51805 7	[30]
Aspergillus oryzae	CBS 102.07T	A. oryzae_Des1	Unknown source	EF661560	EF661506	[30]
	NRRL 458 = ATCC 10063	A. oryzae_Des2	Unknown source	EF661562	EF661507	[30]
Aspergillus parasiticus	CBS 260.67 = DTO 046- C2	A. parasiticus_Jap	Japan	MG662400	MG51801 3	[30]
Aspergillus pipericola	CBS 143680T	A. pipericola_des	Unknown source	MG662385	MG51808 7	[30]
Aspergillus pseudocaelatus	CBS 117616T	A.pseudocaelatus_Itu_Arg	Argentina	EF409242	MG51799 5	[30]
Aspergillus pseudonomius	IBT 12657 = DTO 303-A4	A. pseudonomius_des	Unknown source	MH279418	MG51812 8	[30]
	DTO 267-H7	A. pseudonomius_Tai	Thailand	MH279417	MG51810 2	[30]
Aspergillus sergii	CBS 130017T = DTO 223- C9	A. sergii_Far_Por	Portugal	JF412769	MG51805 9	[30]
Aspergillus subflavus	CBS 143683T = DTO 326- E8	A. subflavus_Mov_Rum	Rumania	MH279429	MG51814 3	[30]
Aspergillus tamarii	DTO 066-A1	A.tamarii_Ind	Indonesia	-	MG51798 8	[30]

Aspergillus vandermerwei	DTO 199-A9 = CBS 129201	A.vandermerwei_Cal_EUA	USA	MH279390	MG518032	[30]
Aspergillus muricatus	NRRL 35674	A. muricatus	Unknown source	EF661434.1	EF661377.1	[30]
Aspergillus flavus	As 2		Paraguay	MN478355	MN443160	This study
Aspergillus flavus	As 5		Paraguay	MN478356	MN443161	This study
Aspergillus flavus	As 7		Paraguay	MN478357	MN443174	This study
Aspergillus flavus	As 8		Paraguay	–	MN443162	This study
Aspergillus flavus	As 10		Paraguay	MN478358	MN443175	This study
Aspergillus flavus	As 21		Paraguay	–	MN443176	This study
Aspergillus flavus	As 32		Paraguay	MN478359	MN443177	This study
Aspergillus flavus	As 33		Paraguay	–	MN443178	This study
Aspergillus flavus	As 34		Paraguay	–	MN416023	This study
Aspergillus flavus	As 38		Paraguay	–	MN443163	This study
Aspergillus flavus	As 41		Paraguay	MN478360	MN443164	This study
Aspergillus flavus	As 54		Paraguay	–	MN443180	This study
Aspergillus flavus	As 69		Paraguay	–	MN443179	This study
Aspergillus flavus	As 70		Paraguay	MN478361	MN443165	This study
Aspergillus flavus	As 76		Paraguay	–	MN443166	This study
Aspergillus flavus	As 78		Paraguay	–	MN443167	This study
Aspergillus flavus	As 79		Paraguay	–	MN443168	This study
Aspergillus flavus	As 84		Paraguay	MN423342	MN393232	This study
Aspergillus flavus	As 88		Paraguay	–	MN393233	This study
Aspergillus flavus	As 93		Paraguay	MN423341	MN416022	This study
Aspergillus flavus	As 96		Paraguay	MN423340	MN416021	This study
Aspergillus flavus	As 101		Paraguay	MN478362	MN443169	This study
Aspergillus flavus	As 103		Paraguay	MN478363	MN443170	This study
Aspergillus flavus	As 118		Paraguay	MN478364	MN443171	This study
Aspergillus flavus	As 138		Paraguay	–	MN443172	This study
Aspergillus flavus	As 271		Paraguay	–	MN443173	This study

Aspergillus flavus	As 17	Paraguay	MN481409	–	This study
Aspergillus flavus	As 22	Paraguay	MN481410	MN604183	This study
Aspergillus flavus	As 25	Paraguay	MN481411	MN604184	This study
Aspergillus flavus	As 27	Paraguay	MN481412	MN604185	This study
Aspergillus flavus	As 29	Paraguay	MN481413	MN604186	This study
Aspergillus flavus	As 35	Paraguay	MN481414	MN604187	This study
Aspergillus flavus	As 42	Paraguay	MN481415	–	This study
Aspergillus flavus	As 43	Paraguay	MN481416	MN604188	This study
Aspergillus flavus	As 98	Paraguay	MN481417	MN604189	This study
Aspergillus luchuensis	As 4	Paraguay	–	MN604190	This study
Aspergillus flavus	As 16	Paraguay	–	MN604192	This study
Aspergillus flavus	As 67	Paraguay	–	MN604193	This study

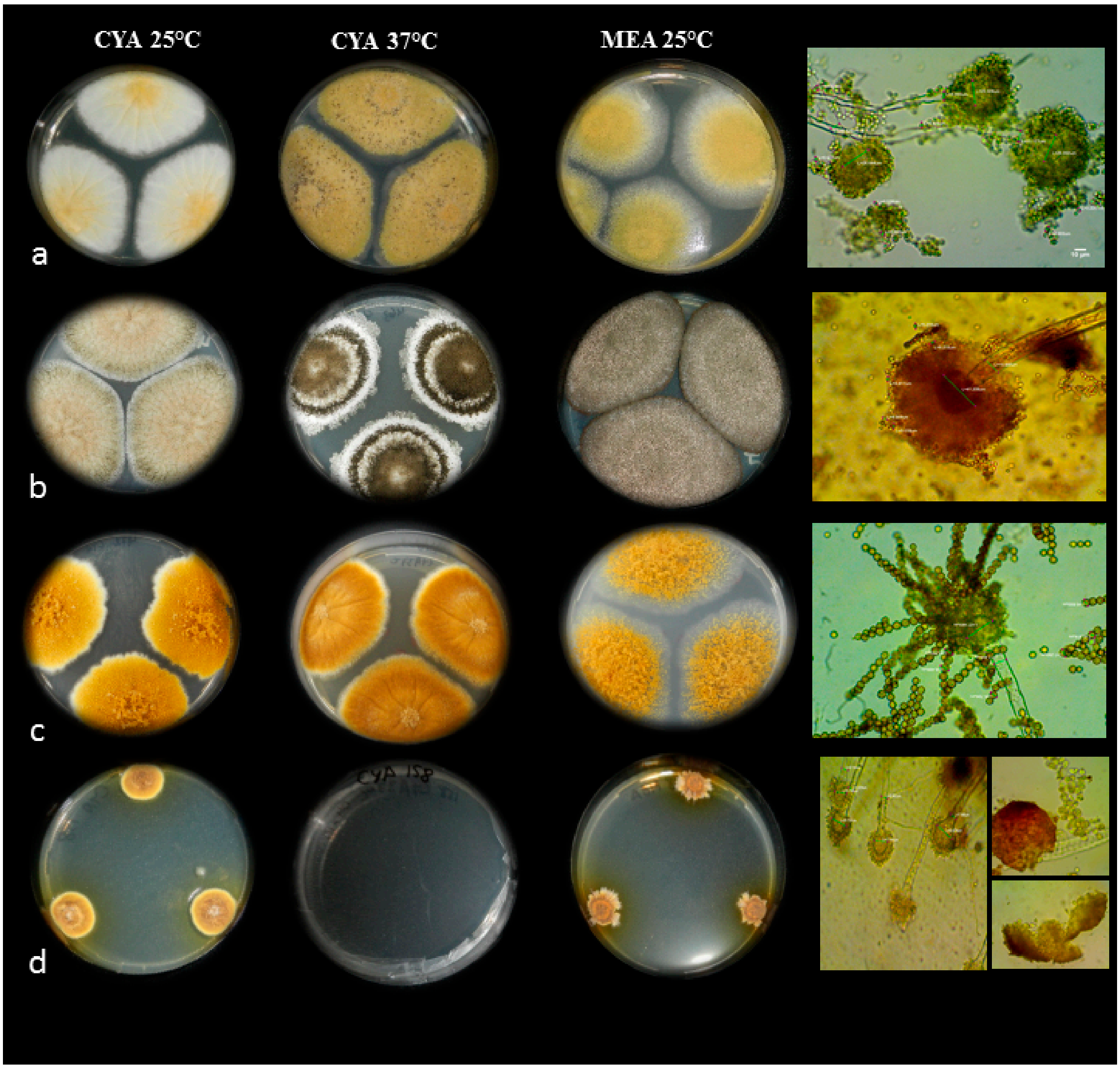

Figure S1: Morphological characteristics (macro- and microscopic) of *Aspergillus* morphotypes. a) *A. flavus* section *Flavi*; b) morphotype *A. awamori* section *Nigri*: c) morphotype *A. tamarii* section *Flavi*; d) morphotype *Aspergillus* section *Aspergillus*. First row: growth in CYA medium at 25 °C, second row: growth in CYA at 37 °C, third row: growth in MEA at 25 °C, fourth row: light microscope image at 40X magnification.

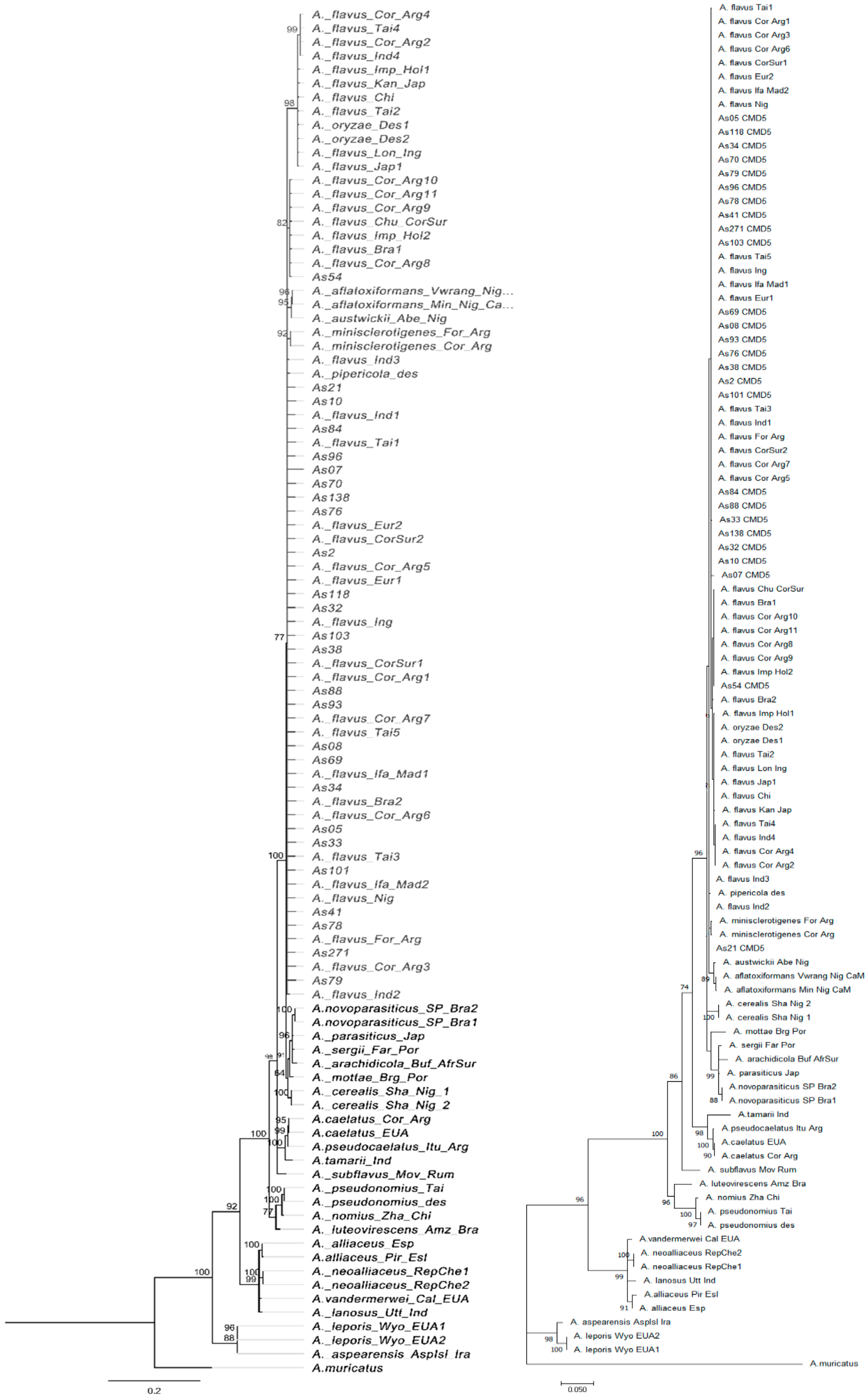

Figure S2. Phylogenetic tree inferred from the partial CaM sequences showing the relationships among members of *Aspergillus flavus*. On the left: phylogenetic tree using the Bayesian Inference based on the Bayesian Markov Chain Monte Carlo (MCMC) or Metropolis-coupled Markov chain Monte Carlo (MCMCMC) model. On the right: maximum likelihood (ML) tree. ML bootstrap support values over 75%, and Bayesian posterior predictive values over 75% are shown above the branches. The tree is rooted with *Aspergillus muricatus* EF661434.1.

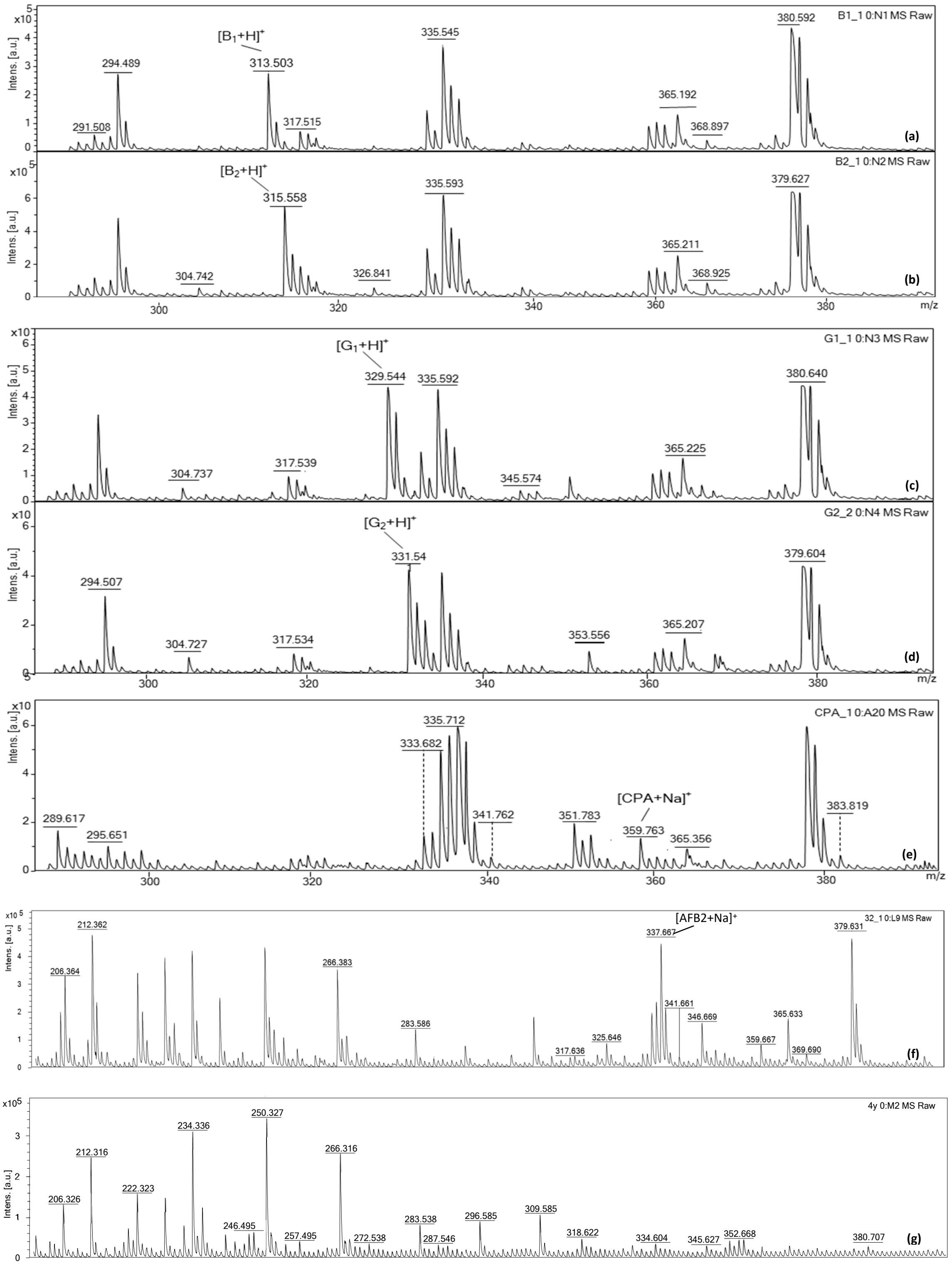

Figure S3: MALDI-TOF MS spectra with standards for aflatoxins B1 (a), B2 (b), G1 (c), G2 (d); Cyclopiazonic Acid (e) and samples positive for AFB2, AS 36 (f) and negative AS 10 (g).
